# Creatine supplementation spares muscle glycogen during high intensity intermittent exercise in rats

**DOI:** 10.1186/1550-2783-7-6

**Published:** 2010-01-29

**Authors:** Hamilton Roschel, Bruno Gualano, Marcelo Marquezi, André Costa, Antonio H Lancha

**Affiliations:** 1 School of Physical Education and Sport, University of São Paulo, São Paulo, Brazil

## Abstract

**Background:**

The effects of creatine (CR) supplementation on glycogen content are still debatable. Thus, due to the current lack of clarity, we investigated the effects of CR supplementation on muscle glycogen content after high intensity intermittent exercise in rats.

**Methods:**

First, the animals were submitted to a high intensity intermittent maximal swimming exercise protocol to ensure that CR-supplementation was able to delay fatigue (experiment 1). Then, the CR-mediated glycogen sparing effect was examined using a high intensity intermittent sub-maximal exercise test (fixed number of bouts; six bouts of 30-second duration interspersed by two-minute rest interval) (experiment 2). For both experiments, male Wistar rats were given either CR supplementation or placebo (Pl) for 5 days.

**Results:**

As expected, CR-supplemented animals were able to exercise for a significant higher number of bouts than Pl. Experiment 2 revealed a higher gastrocnemius glycogen content for the CR *vs*. the Pl group (33.59%). Additionally, CR animals presented lower blood lactate concentrations throughout the intermittent exercise bouts compared to Pl. No difference was found between groups in soleus glycogen content.

**Conclusion:**

The major finding of this study is that CR supplementation was able to spare muscle glycogen during a high intensity intermittent exercise in rats.

## Background

Creatine (CR) plays an important role in rapid energy provision during muscle contraction involving the transfer of the N-phosphoryl group from phosphorylcreatine (PCR) to ADP to regenerate ATP through a reversible reaction catalyzed by phosphorylcreatine kinase (CK). Moreover, Cr is responsible for energy transfer from mitochondria to cytosol. This function is only possible due to the presence of different PCK isoforms linking the sites of ATP generation (i.e., mitochondria; Mt-PCK) to those of ATP consumption (i.e., skeletal muscle and brain; MM-PCK and BB-PCK, respectively) [1,2].

Several studies have focused on the ergogenic capacity of CR loading since its efficacy to increase skeletal muscle CR content in humans has been demonstrated [3]. In fact, a growing body of evidence points out the benefits of CR supplementation in short-term high intensity activities (for review, see [4]), although the mechanisms by which this supplement exerts its effects remains to be fully explored. For instance, the effects of CR supplementation on muscle glycogen content remains to be understood.

Using a one-legged exercise model, it was shown that postexercise muscle glycogen storage can be greater augmented by CR plus carbohydrate supplementation following exercise, as compared to carbohydrate ingestion alone [5]. Lately, these findings have been confirmed by others [6-9]. In addition, it has been demonstrated that carbohydrate supplementation during exhaustive running attenuates the decline in oxidative ATP resynthesis in type I fibres, as indicated by sparing of both PCR and glycogen [10]. However, it is debatable whether CR supplementation is capable of sparing glycogen content during exhaustive exercises. Recently, it was shown that 5-d CR supplementation under conditions of controlled habitual dietary intake had no effect on muscle glycogen content at rest or after continuous endurance exercise [11]. However, it is worth noting that these findings cannot be extrapolated to intermittent exercise, which is knowingly the type of exercise that is the most benefitted by CR supplementation. It is well established that the PCR-CK system plays a crucial role in energy provision during high intensity intermittent exercise. As intramuscular PCR diminishes, the energy provision becomes more reliant on glycolysis (and muscle glycogen) to provide the needed ATP [12-15]. We hypothesized that an increase in PCR content (and in its resynthesis at the rest periods between sets) during intermittent exercise would slow down the PCR decline, followed by less reliance on glycolysis, which would ultimately result in muscle glycogen sparing.

Thus, due to the current lack of clarity, we investigated the effects of CR supplementation on muscle glycogen content after high intensity intermittent exercise in rats. Firstly, we performed an experiment to ensure that CR-supplementation was able to delay fatigue in the adopted exercise protocol. Then, we examined the CR-mediated glycogen sparing effect in intermittent sub-maximal exercise. Assuming that plasma lactate concentration is suggestive of anaerobic pathway flux, we also measured this metabolite throughout the exercise session.

## Methods

### Experiment 1

#### Animals

Sixteen male Wistar rats, weighing 218.14 ± 4.76 g were kept on a normal light/dark cycle in a climate-controlled environment for the duration of the study. The rats were maintained in individual cages and were unable to perform spontaneous exercise. All animals were previously submitted to an anaerobic threshold test, which consisted of a progressive overload swimming test for the anaerobic threshold determination, using external weights attached to the animal's chest [16]. Then, the rats were randomly assigned to either the creatine supplementation group (CR n = 8) or the placebo group (Pl n = 8). Principles of laboratory animal care (NIH publication No. 86-23, revised 1985) were followed, as well as specific national laws (n° 9.605/1998). All procedures were approved by the Ethics Committee of the Biomedical Sciences Institute of the University of São Paulo.

#### Feeding and Supplementation Protocols

Animals were fed *ad libitum *standard chow (Labina, Ralston Purina do Brasil^®^) and water. CR supplementation or placebo (water) was administered via gavage. The researchers were blinded to the treatments. Supplementation protocol consisted of two daily dosages of 300 mg each, for 5 days. We had previously found this protocol to be effective in increasing total CR content by approximately 15% in Wistar rats' gastrocnemius muscle (unpublished data). Moreover, the total amount of CR administered in our supplementation protocol is equal to or even more than those amounts used in other studies that also have shown increased total CR at around 25% [17,18].

#### Experimental Procedure

All animals underwent a 12 h overnight fasting period before the experimental protocol. The animals were weighed immediately prior to exercise, and then the workload utilized during the experimental protocol was determined, accounting for changes in BW. The animals were then submitted to intermittent high-intensity swimming exercise bouts of 30-second duration. The bouts were performed using a 50% higher external load (attached to the rat's chest) than the one correspondent to the anaerobic threshold. Swimming bouts were interspersed by 2-minute rest intervals. Animals were submitted to as many bouts as possible until fatigue. Fatigue was determined when the rat was submerged for longer than 3 seconds.

### Experiment 2

Once it was demonstrated that the proposed CR supplementation protocol had effectively improved time-to-exhaustion in an intermittent high intensity exercise, a second experiment was carried out in order to evaluate whether CR supplementation was able to influence glycogen content and blood lactate concentration in a sub-maximal (fixed number of bouts) intermittent high intensity exercise protocol.

#### Animals

Twenty eight male Wistar rats, weighing 217.55 ± 3.54 g were kept on the same conditions as previously described for experiment 1. The procedures for randomization and group assignment (CR - n = 14; Pl - n = 14), the anaerobic threshold test, feeding and supplementation protocols were also identical to those of experiment 1.

#### Experimental Procedure

All animals underwent a 12 h overnight fasting period before the experimental protocol. They were submitted to 6 bouts of 30-second swimming exercise with supra anaerobic threshold workloads (50% higher than the anaerobic threshold correspondent load). Immediately before testing, animals were weighed and workloads were then calculated. Swimming bouts were interspersed by two-minute rest intervals.

#### Blood and Tissue Collection

Blood samples (25 μl) were drawn from the tail vein at rest, after a ten-minute unloaded warm-up, and at the end of the two-minute recovery period correspondent to each of the 6 swimming bouts. After the last bout, animals were decapitated and soleus and gastrocnemius muscles were extracted, immediately frozen in liquid nitrogen and kept at -70°C for further glycogen analysis.

#### Blood and tissue assays

Blood lactate concentration was assessed by an electrochemical technique (Lactate Analyzer - Yellow Springs Instruments 2300 Stat Plus) after stabilization in sodium fluoride (4.7 mM). Glycogen determination followed a previously described protocol [19]. Fifteen animals from the same group of rats from which experimental groups were selected were used for baseline glycogen determinations.

### Statistical analysis

Results are presented as average ± SD. A Proc Mixed Model (SAS^®^) was performed for blood lactate concentration and glycogen contents [20]. Whenever a significant F-value was obtained, a post-hoc test with a Tukey adjustment was performed for multiple comparison purposes. Correlation between variables was assessed by a Pearson's correlation coefficient Significance level was set at p < 0.05.

## Results

### Experiment 1

#### Number of bouts to exhaustion

**Figure 1:**
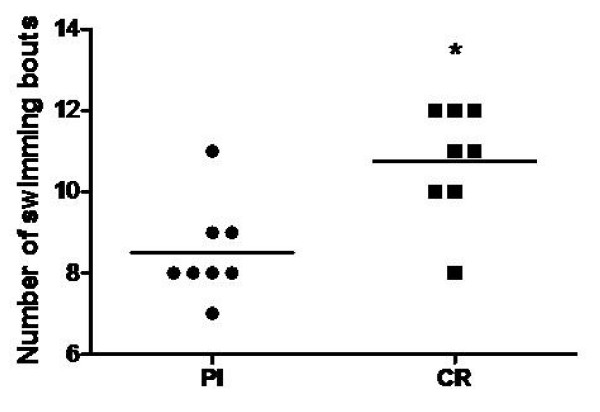
**Effects of creatine supplementation on the number of intermittent high intensity swimming bouts completed until fatigue**. Pl - placebo group; CR - creatine group; * indicates p < 0.05 when compared to Pl group

A significant difference was observed between groups for the number of bouts to exhaustion. Group CR performed a significantly higher (p = 0.035) number of intermittent high intensity swimming bouts than Pl group (10.80 ± 1.67 and 8.42 ± 1.83 respectively) (Figure 1).

### Experiment 2

#### Body weight

Body weight was increased in CR (229.14 ± 4.38 g) when compared to Pl group after the supplementation period (221.71 ± 4.25 g). Additionally, only CR group showed increased body weight when compared to pre supplementation period (217.55 ± 3.54 g).

#### Blood lactate

**Figure 2:**
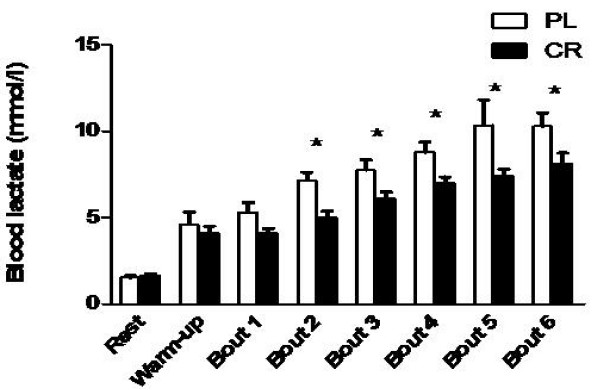
**Effects of creatine supplementation on blood lactate concentrations throughout the experimental protocol (Experiment 2)**. Pl - placebo group; CR - creatine group; * indicates p < 0.05 between groups at the same bout

Blood lactate analysis did not show any differences between groups at rest, after ten-minute unloaded warm-up and after bout 1 of supra anaerobic threshold swimming exercise. However, significantly lower lactate concentrations were observed for CR group in bouts 2, 3, 4, 5 and 6. Figure 2 illustrates blood lactate concentration throughout the experimental protocol.

#### Glycogen content

**Figure 3:**
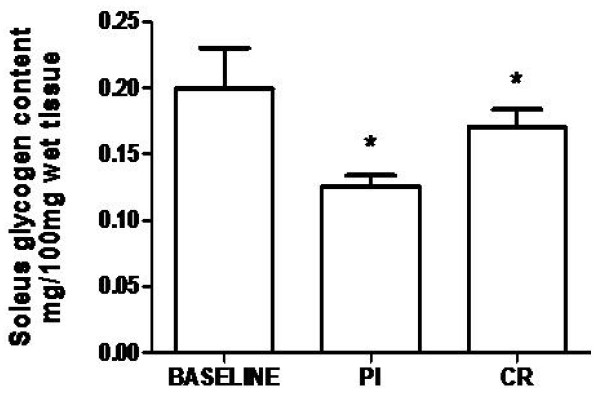
**Effects of creatine supplementation on soleus glycogen content**. Pl - placebo group; CR - creatine group; * indicates p < 0.05 when compared to baseline

Pl and CR groups (0.14 ± 0.03 and 0.17 ± 0.01 mg/100 mg wet tissue, respectively) presented decreased soleus glycogen content compared to baseline (0.19 ± 0.03 mg/100 mg wet tissue). No differences were found between groups (Figure 3).

**Figure 4:**
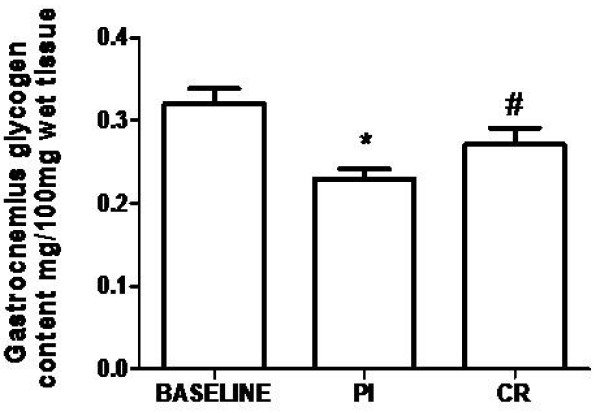
**Effects of creatine supplementation on gastrocnemius glycogen content**. Pl - placebo group; CR - creatine group; * indicates p < 0.05 when compared to baseline. # indicates p < 0.05 when compared to Pl group

A significant interaction was found for gastrocnemius glycogen. CR group showed significant higher glycogen content compared to Pl (33.59%; 0.17 ± 0.01 *vs*. 0.13 ± 0.02 mg/100 mg wet tissue for CR and Pl groups, respectively). Moreover, only Pl group presented a significant decline (39.34%) in glycogen content when compared to baseline values (0.32 ± 0.01 mg/100 mg wet tissue) (Figure 4). Additionally, a significant inverse correlation (p < 0.05 - R= -0.67) between plasma lactate and muscle glycogen level (gastrocnemius) was found.

## Discussion

The aim of this study was to investigate the effects of CR supplementation on muscle glycogen content after high intensity intermittent exercise in rats. The major finding of this study is that a 5-day CR supplementation spared the gastrocnemius but not soleus glycogen content after a sub-maximal intermittent exercise in rats. The decreased blood lactate concentration in CR-supplemented rats supports the notion that the anaerobic glycolytic system has been less utilized as an energy source during the exercise protocol. The CR-induced glycogen sparing might partially explain the improved performance often observed in intermittent exercises as a consequence of this supplement. The absence of significant change in soleus glycogen content is not surprising and reflects the rather low CR and glycogen content in type I vs. type II fibers [21], minimizing the impact of our intervention in the predominantly oxidative soleus muscle.

The possible role of CR supplementation on muscle glycogen modulation has been previously pointed out [5]. The authors demonstrated that postexercise muscle glycogen storage can be augmented by CR and carbohydrate supplementation following exercise compared with carbohydrate ingestion alone. Thereafter, it has been shown that CR-supplemented subjects, during a phase of rehabilitation from immobilization-induced muscle atrophy, had larger muscle glycogen content when compared with non-supplemented subjects (650 versus 520 mmol/kg dry weight) [22]. Accordingly, an 18% increase in muscle glycogen content has been reported as a result of 5 days of concomitant CR and carbohydrate supplementation compared with placebo ingestion [8]. It has been shown that performing a glycogen loading protocol (exhaustive exercise followed by a high carbohydrate diet for 3 days) after CR loading resulted in a 10% greater glycogen content when compared to a glycogen loading before CR loading protocol [6]. In light of these findings, it has been speculated that CR supplementation could beneficially affect performance by modulating pre-exercise muscle glycogen content. Furthermore, it has been speculated that CR loading could also affect performance during exercise by increasing PCR content and consequently decreasing the reliance on glycolysis and muscle glycogen [23,24].

However, no effect of a 5-day CR supplementation on muscle glycogen content has been reported in healthy volunteers either at rest or after continuous endurance exercise to exhaustion [11]. This prompted the investigators to conclude that CR *per se *is not sufficient to alter muscle glycogen content. In the current trial, we noted greater glycogen content in the gastrocnemius muscle following exercise in the 5-day CR supplemented rats, indicating that CR loading is capable of sparing glycogen content throughout an intermittent exercise bout. Some methodological differences between the studies may explain the dissonant findings.

First, the findings obtained with continuous endurance exercise [11] cannot be extended to intermittent exercise. In the latter, it is well established that the ergogenic effect of CR is more pronounced. Since ATP synthesis rate from the creatine kinase reaction with CR loading is reduced dramatically in the first few seconds, rest intervals are crucial to allow adequate (though not complete) aerobic-dependent PCR resynthesis (for details, see [15]). In fact, CR supplementation plays a major role in energy provision during short-duration intermittent exercise; in contrast, energy necessary to maintain long-duration endurance exercise occurs predominantly via aerobic and anaerobic pathways in detriment to the PCR-CR system. In light of this, it is reasonable to speculate that during intermittent exercise, increased muscle PCR content could spare glycogen, serving as an immediate energy source in the myocyte. Accordingly, the lower blood lactate concentration seen in CR group may be a result of a reduced flux through the anaerobic glycolytic pathway or even a shift in glucose metabolism towards oxidation as previously seen in L6 rat skeletal muscle cell [25]. This notion is further supported by the negative relationship between blood lactate concentration and muscle glycogen content observed in the present study. Alternatively, since plasma lactate concentration represents the net result of overall lactate production and utilization by the tissues, it is possible that an increase in tissue lactate utilization could have also accounted for the lower plasma lactate concentration observed in the CR group.

Second, it is not possible to rule out that the discordant findings are a result of different experimental models investigated. Previous studies have demonstrated major differences between species regarding CR transport, bioavailability, metabolism, uptake and physiological response, as previously pinpointed by others [26,27]. For instance, a rapid and nearly complete gastrointestinal absorption of CR has been shown in humans [3], contrasting with the lack of absorption in an herbivorous animal such as the horse. In addition, an elegant study [27] highlighted the species-and tissue-specific response to CR intake. The authors demonstrated that CR administration can induce chronic hepatitis in mice, but not in rats, suggesting large variance even between close species. Furthermore, in contrast to humans, muscle glycogen content may have little effect on endurance during submaximal exercise in mice [28], although, the authors emphasize that muscle glycogen might have a greater impact on performance under anaerobic muscular activity, as in the case of the present study. Therefore, taking into account the species-specific differences, the current findings should be further validated and cannot be fully extrapolated to humans at this point.

Although we did not measure muscle CR content, we believe that the adopted supplementation regime has efficiently increased intramuscular CR based on previous data from our laboratory and the results of others that have used similar protocols [17,18]. Moreover, the rapid increase in body weight observed only in CR group suggests that creatine uptake occurred since water retention is a well documented effect of CR supplementation [4]. However, we acknowledge that the lack of muscle CR assessment could be viewed as a limitation of the present study. Still, one may argue that the lack of resting glycogen measurement after CR supplementation could be considered a factor in this study because it would preclude dissociating the effect of CR on glycogen content during exercise from that at rest. However, accumulative evidence indicates that CR supplementation, in the absence of prior exercise, does not increase muscle glycogen storage [5]. Recently, convincing findings that dietary CR supplementation does not influence resting muscle glycogen content in recreationally active volunteers has been provided, supporting the hypothesis that dietary CR-associated increases in muscle glycogen content are a result of an interaction between dietary supplementation and other mediators of muscle glucose transport, such as muscle contraction [11]. Accordingly, we also showed that CR supplementation (the same protocol used in the current study) does not increase glycogen content in sedentary Wistar rats [29]. Therefore, the fact that the rats were non-exercised in the present study allows assuming that the sparing effects of CR on glycogen content occurred during exercise. Another possible debatable point is the lack of a control group receiving isonitrogenous and isoenergetic diet. However, this is unlikely to play a role in the results, since several studies have shown creatine-induced glycogen accretion even when compared with a carbohydrate supplemented group [6-9]. Finally, it is worth emphasizing that rats were submitted to 12-h fasting before exercise, and muscle glycogen contents were rather lower than those reported by others [30-34]. Nonetheless, the rats were submitted to a normal light/dark cycle. Considering that rats usually feed during dark and sleep during light, the 12 h-food restriction during dark cycle prior to the exercise reflects a "real" fasting closer to 24 hours and not 12 hours. For this reason, we can assume that the longer than usual fasting period in this study can partially explain the low muscle glycogen observed. Thus, the current findings cannot be extrapolated to a "glycogen loaded" condition (i.e., post absorptive state or after carbohydrate supplementation). However, considering that individuals engaged in intermittent sport modalities achieve partial glycogen depletion in the closing minutes of a competition or training session, the findings of this study still have importance for those desiring to enhance sport performance.

## Conclusions

We demonstrated that CR supplementation is able to spare gastrocnemius glycogen content and reduce blood lactate concentration in rats submitted to intermittent high intensity exercise. If confirmed by human studies, CR-induced glycogen sparing could be another mechanism to explain the ergogenic effect of CR supplementation in intermittent exercise.

## Competing interests

The authors declare that they have no competing interests.

## Authors' contributions

All authors have read and approved the final manuscript. HR is the principal investigator of the project. HR, BG and AHLJ designed the study; HR, MM and AC collected the data; BG and HR conducted data analysis; HR, BG and AHLC wrote the manuscript.
